# Time-course analysis of *Drosophila suzukii* interaction with endoparasitoid wasps evidences a delayed encapsulation response compared to *D*. *melanogaster*

**DOI:** 10.1371/journal.pone.0201573

**Published:** 2018-08-02

**Authors:** Alessia Iacovone, Nicolas Ris, Marylène Poirié, Jean-Luc Gatti

**Affiliations:** Université Côte d’Azur, INRA, CNRS, Institut Sophia Agrobiotech, Sophia Antipolis, France; Universita degli Studi della Basilicata, ITALY

## Abstract

*Drosophila suzukii* (the spotted-wing Drosophila) appears to be unsuitable for the development of most Drosophila larval endoparasitoids, be they sympatric or not. Here, we questioned the physiological bases of this widespread failure by characterizing the interactions between *D*. *suzukii* and various parasitoid species (*Asobara japonica*, *Leptopilina boulardi*, *Leptopilina heterotoma* and *Leptopilina victoriae*) and comparing them with those observed with *D*. *melanogaster*, a rather appropriate host. All parasitoids were able to oviposit in L1 and L2 larval stages of both hosts but their propensity to parasitize was higher on *D*. *melanogaster*. *A*. *japonica* and, to a much lesser extent, *L*. *heterotoma*, were the two species able to successfully develop in *D*. *suzukii*, the failure of the parasitism resulting either in the parasitoid encapsulation (notably with *L*. *heterotoma*) or the host and parasitoid deaths (especially with *L*. *boulardi* and *L*. *victoriae*). Compared to *D*. *melanogaster*, encapsulation in *D*. *suzukii* was strongly delayed and led, if successful, to the production of much larger capsules in surviving flies and, in the event of failure, to the death of both partners because of an uncontrolled melanization. The results thus revealed a different timing of the immune response to parasitoids in *D*. *suzukii* compared to *D*. *melanogaster* with a lose-lose outcome for parasitoids (generally unsuccessful development) and hosts (high mortality and possible reduction of the fitness of survivors). Finally, these results might suggest that some European endoparasitoids of Drosophila interact with this pest in the field in an unmeasurable way, since they kill their host without reproductive success.

## Introduction

The spotted-wing Drosophila, *Drosophila suzukii* Matsumura (Diptera: Drosophilidae), is a Southeast Asian species of the *melanogaster* subgroup that has expanded rapidly in North-America and Europe since its first record in 2008 [1–3]. Unlike most fruit flies, *D*. *suzukii* has a modified ovipositor that allows egg laying through the skin of ripening fruits [4,5]. Damages are mainly due to larval feeding on the fruit pulp and pathogenic infections developing at the prick. *D*. *suzukii* is highly polyphagous, larvae developing in a wide range of fruit crops—mainly red fruits (raspberry, strawberry) and stone fruits (cherry, apricot, plum)—as well as wild fruits which act as reservoirs [6–8]. The control of this pest mainly relies on the use of chemical insecticides that increases production costs, results in the presence of residues in harvested fruits, and threaten biodiversity. The development of biological control methods is thus highly desirable in newly invaded countries. However, the use of natural enemies, such as parasitoid wasps, requires a thorough knowledge of their behavioral and physiological interactions with the fly.

Hymenopteran parasitic wasps lay eggs on (ectoparasitoids) or inside (endoparasitoids) arthropod hosts’ larvae or pupae that will be consumed by the developing parasitoid larva. Several endoparasitoid species are well known to use drosophila as hosts, including *D*. *melanogaster*, regulating their field populations [9,10]. The main fly immune response to endoparasitoids is encapsulation *i*.*e*. the deposit of several layers of specialized immune cells (plasmatocytes and lamellocytes) around the parasitoid egg, together with the activation of the phenoloxidase cascade, leading to the formation of a melanized capsule [11,12]. Most *Drosophila* parasitoid species nevertheless prevent encapsulation and regulate the host physiology by injecting venom along with the eggs [13–17]. Therefore, the outcome of parasitism is mainly determined by the interaction between the fly immune defense and the wasp venom virulence factors.

*D*. *suzukii* interaction with parasitoid wasps has recently received increased interest with a view of developing the biological control for this pest. Identified species reported to be associated with the fly in Asia include *Asobara japonica* (Braconidae), *Ganaspis* species (Figitidae), *Leptopilina japonica* (Figitidae) and pupal parasitoids from the *Trichopria* genus (Diapriidae) [18–26]. These parasitoids have a wide host range except the Asian *Ganaspis* species. In Europe and USA, the only species reported to successfully parasitize *D*. *suzukii* are pupal parasitoids, *Trichopria drosophilae* and *Pachycrepoideus vindemmiae* (Pteromalidae) [27–32]. Larval endoparasitoids of drosophilids, such as *Leptopilina boulardi* and *L*. *heterotoma*, were reported to be unsuccessful to reproduce on *D*. *suzukii* in laboratory conditions, despite a high infestation rate [27]. This strong *D*. *suzukii* resistance was proposed to result from the high hemocyte load in this species [28,29] since this trait was previously correlated with the percentage of successful encapsulation in species of the *melanogaster* subgroup [33]. Yet, the time-course and features of encapsulation of the different parasitoid strains (the term “strains” encompassing here and below both inter- and intra-specific variation) by *D*. *suzukii* have never been analyzed.

Here, we have investigated in more detail the behavioral and physiological interactions between *D*. *suzukii* and several larval parasitoid strains, either allopatric (European origin) or sympatric (Asiatic origin) to the fly. We first evaluated the acceptance and suitability of the fly larval stages for each parasitoid wasp and characterized the outcome of each interaction. Then, we analyzed the time-course of the host immune response to the parasitoid. Results identify precisely when parasitism fails and raise the question of the underlying processes. In addition to the variety of physiological interactions between larval endoparasitoids and *D*. *suzukii*, we highlight striking differences between *D*. *suzukii* and *D*. *melanogaster* in the establishment and time-course of the immune response to parasitoids, with a “delay” in the encapsulation response in the invasive Asian fly.

## Materials and methods

### Insect strains and rearing

The *D*. *suzukii* strain, obtained from a population collected in Sainte-Foy-lès-Lyon (Rhône, France), was kindly provided by Dr. R. Allemand (LBBE, University Lyon 1, France). The *D*. *melanogaster* strains Nasrallah (Gif stock no. 1333) and YR (Gif stock no. 1088) are respectively susceptible and resistant to the ISy strain of *Leptopilina boulardi* [34]. All flies laid eggs on standard *Drosophila* corn meal-yeast-agar-nipagin medium at 25 °C, and the vials were then transferred to 20 °C (12:12 L: D photoperiod).

The *L*. *boulardi* ISy (Lby, Gif stock no. 486) and ISm (Lbm, Gif stock no. 431) isofemale lines were previously described [35]. Briefly, Lby and Lbm were obtained from populations of Brazzaville (Congo) and Nasrallah (Tunisia), respectively. The *L*. *boulardi* strain Lb_16_ was founded in February 2014 from a field population collected in Dordogne (France). The European strain of *L*. *heterotoma* (Lh_Goth_, Gif stock no. 548) was obtained from Gotheron (France). The Japanese strains of *L*. *heterotoma* (Lh_Japan_) and *L*. *victoriae* (Lv) (described in [22]), were provided by Pr. M. T. Kimura (Hokkaido University, Japan). The thelytokous Japanese strain of *Asobara japonica* (Aj) [20] was kindly provided by the BIPE laboratory (University of Picardie-Jules Verne, France). All strains, except Lb_16_, had been maintained under laboratory conditions for several years at the time of the experiments. Parasitoids were reared on the susceptible *D*. *melanogaster* Nasrallah strain at 25 °C (12:12 L: D), 50–60% humidity. Emerged adult wasps were kept at 20 °C on agar medium with water and honey. Experiments were performed using 3 to 10 days-old females.

### Oviposition behavior

*D*. *suzukii* females start laying eggs 72–96 hours after emergence and lay less than 10 eggs per day at 20–21 °C. For parasitism experiments, larvae were thus produced by transferring ten to twenty 5 days-old mated females into new vials for 96h (L1) or 120h (L2), respectively. L1 or L2 larvae were then collected manually. *D*. *melanogaster* mated adult flies (72-96h old) could lay eggs for 4h, and larvae were collected 48h (L1) and 72h (L2) later, respectively. The duration of the development stages was roughly similar for *D*. *suzukii* and *D*. *melanogaster* under our rearing conditions, as reported previously [36].

Host parasitism assays were performed in 24 mm (Ø) plastic dishes containing a 3–4 mm layer of standard medium at 25 °C. In each experiment, 20 *D*. *suzukii* or *D*. *melanogaster* L1 or L2 were exposed to a single naive mated female wasp during 4h. Fly larvae were dissected 48h later to evaluate the wasp propensity to parasitize (PP: proportion of female wasps having oviposited at least one egg) and the infestation rate (IR: percentage of successfully infested hosts whatever their status, dead or alive, or the wasp status, free floating or encapsulated egg, free, partially or completely encapsulated larva). The super-parasitism rate was consistently low (0–6%; mean of 2%) and did not significantly differ between host species. Super-parasitized larvae were thus considered to estimate the infestation rate but not to evaluate the parasitoid encapsulation rate.

### Host suitability

Three criteria of host suitability—acceptability for oviposition, development success of the parasite larvae and encapsulation rate—were evaluated as follows. Three pools of 20 to 30 larvae (n_l_) were independently offered in a vial to batch of 3 female wasps of a given strain during 24 hours at 25 °C. The parasitoid offspring number (n_p_) and the number of emerged flies (n_a_) were counted for each replicate. Emerged flies were then dissected to evaluate the number of hosts containing an encapsulated parasitoid (capsule) (n_c_). Five control vials were used in parallel to estimate the median survival rate (s) of *D*. *suzukii* in the absence of parasitoids.

Two parameters were then calculated for each assay:

- The success of parasitism (SP) defined as the ratio between the number of emerged adult wasps and the estimated number of infested hosts [37]:
  $$SP\;=\;\frac{n_{p}}{n_{l}-(\frac{n_{a-n_{c}}}{s})}$$
- The encapsulation rate (ER) defined as the ratio between the number of hosts containing a capsule and the estimated number of infested hosts:
  $$ER\;=\;\frac{n_{c}}{n_{l}-(\frac{n_{a-n_{c}}}{s})}$$

These two estimates are valid under the assumption that encapsulation is the sole mechanism of host resistance to parasitoids.

### Monitoring host-parasitoid immune interaction

A qualitative analysis was first performed to describe the time-course of the immune interaction of all the wasp strains with *D*. *suzukii*. To do so, we dissected *D*. *suzukii* L1 and L2 larvae 24, 48, 72, 96, 168 and 240 h after parasitism. As a control, we parasitized the *D*. *melanogaster* YR strain by the *L*. *boulardi* ISy line since the outcome of this interaction is the encapsulation of the parasitoid [38]. Based on this analysis, we identified three main types of interaction outcome: survival of the parasitoid with no encapsulation, death of both partners, and encapsulation. For a more detailed analysis of the interaction outcome, pools of 20 *D*. *suzukii* L1 or L2 were exposed to parasitism and dissected 48h (L1 and L2) or 72h later (only L1 since L2 larva were at the pre-pupal stage at that time). The percentages of alive or dead fly larvae containing free floating egg, free parasitoid larva, wasp egg or larva only surrounded by a thin coat of lightly-colored cells or with a few black spots (partial melanization), encapsulated eggs, and completely encapsulated parasitoid larva (fully melanized) were calculated.

### Statistical analysis

Data on oviposition behavior and host suitability were analyzed using GLM with a binomial distribution of response variables (proportions). In all cases, a full model was used, including all possible explanatory variables (Parasitoid strain, Female experience, Host species and/or Host stage) and, when possible, their interactions (S1 File). A backward procedure was applied to select the most relevant model according to the Akaike Information Criterion (AIC), the distribution of the residuals being checked visually. An analysis of deviance was then performed to test for the influence of the selected explanatory variables and their interactions. Finally, post-hoc tests were performed in some cases using the Tukey HSD test. All the procedure was performed with the R software (http://www.R-project.org), its graphical interface “R commander” and related packages (in particular “MASS” and “multcomp”).

## Results

### Host acceptance

#### Propensity to parasitize (PP)

First, we evaluated the propensity of naive or trained female wasps to oviposit on L1 or L2 larvae of *D*. *suzukii* and *D*. *melanogaster* (propensity to parasitize, PP; Table 1 and S1 File). Data were analyzed using a full statistical model including all explanatory variables: “Host species”, “Host stage”, “Parasitoid strain”, and “Female training” (i.e. female parasitoids were individually allowed to previously parasitized the same number of hosts) but the “Host stage” variable was not kept in the model. Two variables, “Host species” (χ^2^_1df_ = 6.9; p = 0.008) and “Parasitoid strain” (χ^2^_6df_ = 69.9; p<10^−3^) had a significant effect. On average, the parasitoids PP was higher on *D*. *melanogaster* (87%) than *D*. *suzukii* (75%). *Asobara japonica* was the least “motivated” parasitoid with a lower PP than all other species/strains except *Leptopilina victoriae*. *L*. *heterotoma* Gotheron (Lh_Goth_) and *L*. *boulardi* Lbm had a higher PP but it only significantly differed from that of *L*. *victoriae*. Finally, the “Female training” variable had no effect (S1 File; χ^2^_1df_ = 2.6; p = 0.108). Surprisingly, trained *A*. *japonica* females were reluctant to parasitize (PP = 0) in three out of the four Host species x Host stage combinations (Table 1).

**Table 1:** Parasitoid propensity to parasitize (PP) and infestation rate (IR).

		*D*. *melanogaster*										*D*. *suzukii*								
HOST STAGE		L1					L2					L1					L2			
		n	PP	IR			n	PP	IR			n	PP	IR			n	PP	IR	
PARASITOID				mean	sd				mean	sd				mean	sd				mean	sd
**Aj**																				
** naive**		8	0.50	0.62	0.16		9	0.22	0.95	0.00		11	0.36	0.40	0.20		23	0.39	0.66	0.24
** trained**		3	0.00	_	_		2	1.00	0.13	0.13		3	0.00	_	_		3	0.00	_	_
**Lv**																				
** naive**		4	1.00	0.80	0.29		4	0.75	0.76	0.21		10	0.50	0.56	0.36		8	0.25	0.80	0.05
** trained**		2	1.00	0.96	0.01		3	1.00	0.95	0.07		8	0.25	0.95	0.05		3	1.00	0.94	0.09
**LhGoth**																				
** naive**		6	0.67	0.92	0.05		4	1.00	0.65	0.06		10	0.90	0.81	0.07		4	1.00	0.91	0.09
** trained**		3	1.00	0.92	0.02		3	1.00	0.95	0.04		3	1.00	0.95	0.00		3	1.00	0.62	0.27
**LhJapan**																				
** naive**		4	1.00	0.54	0.26		4	0.75	0.48	0.09		18	0.67	0.47	0.21		5	0.60	0.41	0.18
** trained**		3	1.00	0.19	0.01		2	1.00	0.71	0.13		4	1.00	0.64	0.13		3	1.00	0.30	0.21
**Lbm**																				
** naive**		4	1.00	0.84	0.13		4	1.00	0.76	0.22		10	0.80	0.41	0.27		4	1.00	0.55	0.16
** trained**		3	1.00	0.87	0.12		3	1.00	0.98	0.02		3	1.00	0.75	0.08		3	0.67	0.19	0.10
**Lby**																				
** naive**		4	0.75	0.79	0.08		4	0.75	0.60	0.28		10	0.60	0.51	0.33		4	0.75	0.22	0.18
** trained**		3	1.00	0.83	0.09		3	1.00	0.87	0.10		4	1.00	0.71	0.07		2	1.00	0.23	0.07
**Lb16**																				
** naive**		3	1.00	0.49	0.07		2	1.00	0.85	0.05		6	1.00	0.58	0.16		5	0.80	0.51	0.29
** trained**		3	1.00	0.90	0.06		3	1.00	0.98	0.03		2	1.00	0.92	0.03		2	1.00	0.97	0.03

Aj, *Asobara japonica*; Lv, *Leptopilina victoriae*; LhGoth, *L*. *heterotoma* Gotheron; LhJapan, *L*. *heterotoma* Japanese strain; Lbm, *L*. *boulardi* ISm strain; Lby, *L*. *boulardi* ISy strain; L16, *L*. *boulardi* strain 16. sd, standard deviation.

#### Infestation rate (IR)

Parasitoid infestation rates were calculated from all females that initiated oviposition (Table 1 and S1 File). The statistical analysis was performed using naive parasitoid females only, due to the reluctance of trained *A*. *japonica* to parasitize. The selection of the model and the analysis of deviance evidenced a complex situation with, notably, a significant triple interaction between the “Parasitoid strain”, the “Host species” and the “Host stage” (χ^2^_6df_ = 48.1; p<10^−3^). This interaction was also significant when considering only the three parasitoids that were sympatric to *D*. *suzukii* in the native area (*A*. *japonica*, *L*. *victoriae*, *L*. *heterotoma* Japan). This suggests that each parasitoid species has its own specificity with regard to the host species and/or stage (χ^2^_2df_ = 9.4; p = 0.009). Interestingly, *A*. *japonica* and *L*. *victoriae*, the two species with the lowest propensity to parasitize, had fairly high infestation rates on *D*. *suzukii*, similar to other parasitoid species.

### *D*. *suzukii* suitability as a host

#### Overall parasitism success

*A*. *japonica*, and to a lesser extent, *L*. *heterotoma* (Lh_Goth_ and Lh_Japan_) were the only species that produced offspring from *D*. *suzukii* (Table 2; S1 Fig). Accordingly, the “Parasitoid strain” variable was highly significant (χ^2^_6df_ = 40.5; p<10^−3^) while the “Host stage” (L1 *vs* L2 larvae) was not kept in the final model (S1 File). We estimated that 41% and 55% of *A*. *japonica* eggs successfully developed in mono-parasitized *D*. *suzukii* L1 and L2 larvae, respectively, the alternate outcome being the death of both partners.

**Table 2:** Suitability of *D*. *suzukii* L1 and L2 larval stages for the parasitoid species/strains.

HOST STAGE		L1						L2				
PARASITOID		Ability	Offspring mean (sd)	Success mean (sd)	Sensitivity	Encapsulation mean (sd)		Ability	Offspring mean (sd)	Success mean (sd)	Sensitivity	Encapsulation mean (sd)
**Aj**		4/4	6.8 (3.0)	0.41 (0.07)	0/4	0.00 (0.00)		4/4	10.3 (2.4)	0.55 (0.10)	0/4	0.00 (0.00)
**Lv**		0/3	0.0 (0.0)	0.00 (0.00)	3/3	0.29 (0.16)		0/3	0.0 (0.0)	0.00 (0.00)	3/3	0.16 (0.19)
**LhGoth**		1/3	0.3 (0.6)	0.01 (0.02)	3/3	0.42 (0.25)		1/3	1.0 (1.7)	0.04 (0.06)	3/3	0.70 (0.15)
**LhJapan**		3/3	1.3 (0.6)	0.07 (0.04)	3/3	0.36 (0.18)		2/3	1.7 (2.9)	0.06 (0.10	3/3	0.41 (0.23)
**Lbm**		0/3	0.0 (0.0)	0.00 (0.00)	3/3	0.09 (0.02)		0/3	0.0 (0.0)	0.00 (0.00)	3/3	0.06 (0.02)
**Lby**		0/4	0.0 (0.0)	0.00 (0.00)	4/4	0.24 (0.20)		0/3	0.0 (0.0)	0.00 (0.00)	3/3	0.30 (0.20)
**Lb16**		0/3	0.0 (0.0)	0.00 (0.00)	3/3	0.08 (0.05)		0/3	0.0 (0.0)	0.00 (0.00)	3/3	0.30 (0.05)

Aj: *Asobara japonica*; Lv, *Leptopilina victoriae*; LhGoth, *L*. *heterotoma* Gotheron; LhJapan, *L*. *heterotoma* Japanese strain; Lbm, *L*. *boulardi* ISm strain; Lby, *L*. *boulardi* ISy strain; Lb16, *L*. *boulardi* strain 16. **Ability**: ability to develop in *D*. *suzukii* (number of successful females/number of tested females); **Offspring**: mean offspring number per female; **Success**: mean parasitism success per female; **Sensitivity**: sensitivity to encapsulation by *D*. *suzukii* (number of females with encapsulated offspring/number of tested females); **Encapsulation**: mean encapsulation rate. sd; standard deviation.

#### Encapsulation rate

The sensitivity to encapsulation was significantly influenced by the parasitoid strain (χ^2^_6df_ = 44.6; p<10^−3^; see S1 File), *A*. *japonica* being the only one not to be encapsulated at all (Table 2). When this species was discarded, the encapsulation rate (ER) still differed among parasitoid strains (S1 File; χ^2^_5df_ = 114.5, p<10^−3^). To a lesser extent, ER was also influenced by the host stage (χ^2^_1df_ = 5.7, p = 0.02), this parameter being globally higher for L2 host larva compared to L1 (S1 File; see also S1 Fig). This allowed identifying three different parasitoid groups: (i) *L*. *boulardi* strains Lbm and Lb_16_ with a low encapsulation rate (<30%) and no emergence; (ii) *L boulardi* Lby strain and *L*. *victoriae* with an intermediate encapsulation rate and no emergence (iii) *L*. *heterotoma* strains Lh_Japan_ and Lh_Goth_ with a high encapsulation rate (36–70%) and sporadic emergence of adult offspring (< 10%).

Parasitized *D*. *suzukii* experienced mortality in all interactions although with variation between replicates. We observed a high mortality rate when flies were parasitized by *L*. *boulardi* and *L*. *victoriae* (mean of 80%) compared to those attacked by *L*. *heterotoma* or *A*. *japonica* (mean of 45%) (S1 Fig).

#### Time-course of the encapsulation response

**Fig 1:**
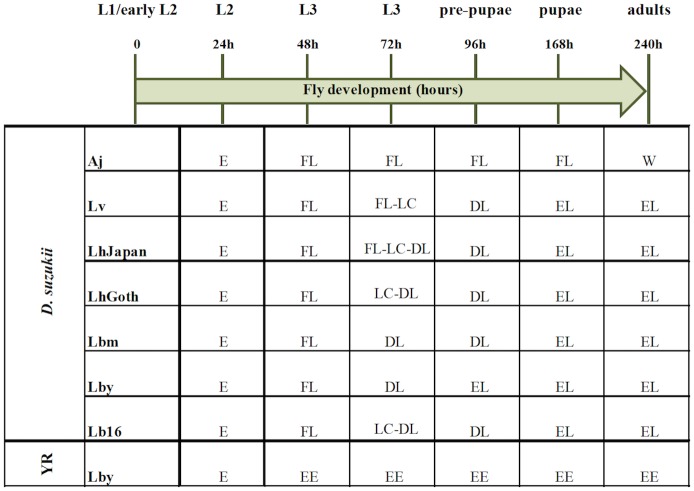
Time-course of the wasp-host interaction in parasitized *D*. *suzukii* larvae. Live larvae of *D*. *suzukii* parasitized by the different parasitoid strains or *D*. *melanogaster* YR (YR) parasitized by *L*. *boulardi* ISy were dissected at different time (0h-240h) and the main observed steps of the encapsulation response are reported. E, free parasitoid egg; EE, encapsulated parasitoid egg; FL, free parasitoid larva; LC, free parasitoid larva with a thin coat of light-colored cells; DL, dead parasitoid larva; EL, encapsulated parasitoid larva; W, developing wasp. Aj, *Asobara japonica*; Lv, *Leptopilina victoriae*; LhGoth, *L*. *heterotoma* Gotheron; LhJapan, *L*. *heterotoma* Japanese strain; Lbm, *L*. *boulardi* ISm strain; Lby, *L*. *boulardi* ISy strain; Lb16, *L*. *boulardi* strain 16.

**Fig 2:**
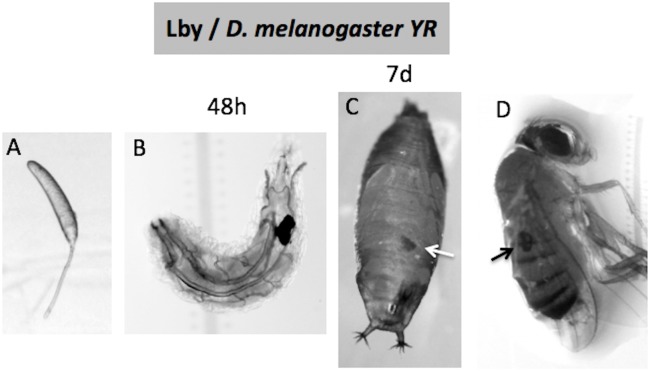
Encapsulation of *L*. *boulardi* ISy (Lby) eggs by the *D*. *melanogaster* YR strain. *D*. *melanogaster* flies were observed and/or dissected at different times after parasitism: (A) after two hours, only free parasitoid eggs were recovered; (B) after 48h, almost all larvae contained an encapsulated egg. The capsules can be observed in the pupae 7 day (7d) post-parasitism (C) and in newborn flies (D) (arrows).

The qualitative time-course of the interaction (parasitism of L1/early L2 *D*. *suzukii* hosts) evidenced clear differences between parasitoid strains (Fig 1). The establishment of the immune response also differed from what was observed in the resistant *D*. *melanogaster* YR control (L1 or L2 flies parasitized by Lby), i.e. the formation of a complete melanized capsule as earlier as 48h post-parasitism [38] (Fig 2).

**Fig 3:**
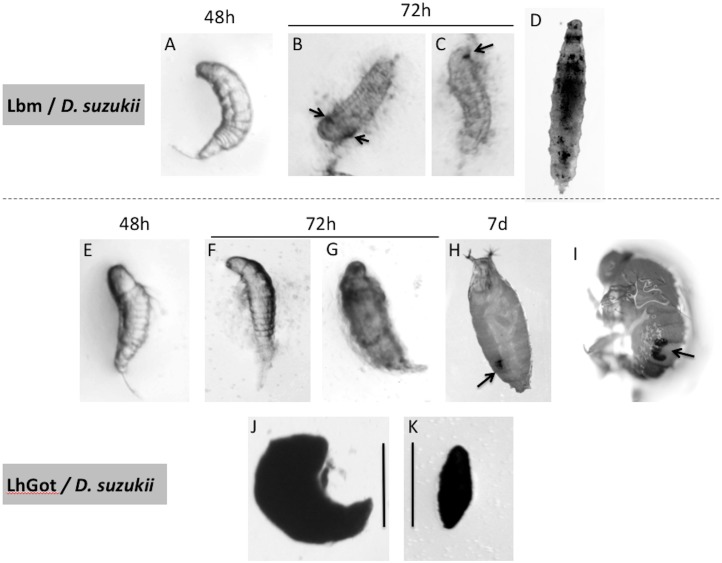
Timing of encapsulation of *L*. *boulardi* Ism (Lbm) and *L*. *heterotoma* Gotheron eggs in *D*. *suzukii* larvae. Host larvae were observed and/or dissected at different times following parasitism by *L*. *boulardi* ISm (Lbm) (A-D) or *L*. *heterotoma* Gotheron (E-I). 48h post-parasitism, free-living larvae were mainly observed for the two parasitoids (A and E). At 72h, Lbm larvae were entangled in a thin layer of cells, and a few melanization spots (arrows) were observed (B, C), whereas only a few cells were found on *L*. *heterotoma* larvae, without melanization (F, G). Most larvae parasitized by Lbm showed an over-melanization response, and the fly was unable to pupate and died (D). Surviving larvae parasitized by *L*. *heterotoma* continued to develop and pupae (H) and emerged adult flies contained a capsule (I). The size of the capsule formed by *D*. *suzukii* against *L*. *heterotoma* (J) and by *D*. *melanogaster* YR against *L*. *boulardi* ISy (Lby) (K) are compared. Bar is 0.2 mm.

In *D*. *suzukii*, the immune response was only noticeable 72h post-parasitism (Fig 1). Three different outcomes were observed depending on the parasitoid species:

1. the absence of any visible host reaction leading to either the death of both partners or the successful development of *A*. *japonica*;
2. the hatching of most *L*. *heterotoma* eggs and surrounding of larvae by a thin coat of lightly colored cells starting 72h post-oviposition (Fig 3). Most parasitoid larvae were dead 96h post-oviposition and complete melanized capsules were observed at 168h. A small percentage of larvae (<10%) avoided encapsulation and successfully developed;
3. the hatching of most *L*. *boulardi* and *L*. *victoriae* eggs followed by the rapid death of the parasitoid larvae and their encapsulation 96h post-parasitism (Lby) or later (Lbm, Lb_16_) (see example for Lbm in Fig 3), leading to a high mortality rate at pupation.

**Fig 4:**
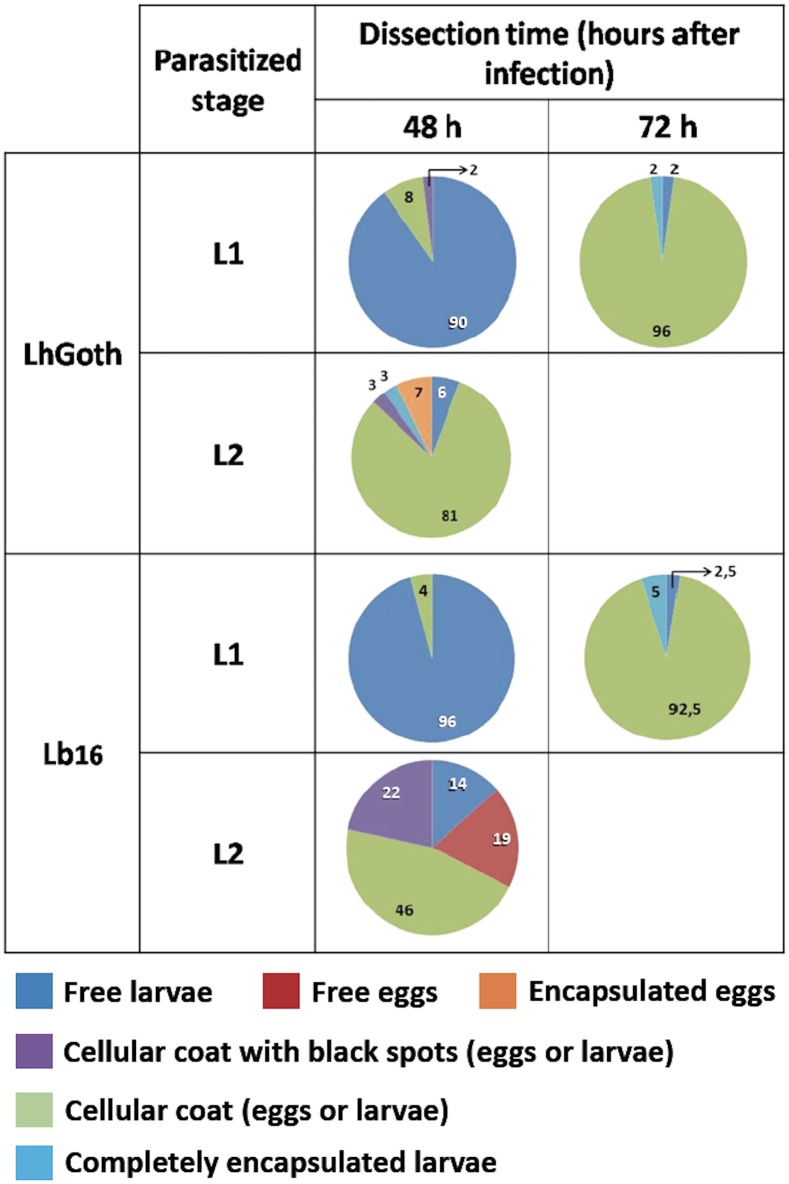
Analysis of *D*. *suzukii* physiological interaction with *L*. *heterotoma* and *L*. *boulardi* parasitoids. *D*. *suzukii* L1 and L2 larvae parasitized by the French strain of *L*. *heterotoma* (Lh_Goth_) or the field strain of *L*. *boulardi* (Lb_16_) were dissected 48h (L1 and L2) or 72h (L1) post-parasitism to evaluate the state of the immune response, as described in Fig 3. Pie charts give the percentage of alive or dead fly larvae containing free parasitoid larvae, free floating eggs, wasp eggs or parasitoid larvae only surrounded by a thin coat of lightly-colored cells, wasp eggs or larvae partially melanized (coat of lightly-colored cells with a few black spots), completely encapsulated parasitoid larvae (fully melanized) and completely encapsulated eggs.

A more detailed and quantitative analysis was performed by dissecting parasitized L1 larvae 48h and 72h post-parasitism, and parasitized L2 larvae 48h post-parasitism. We focus here on the *D*. *suzukii* encapsulation response against Lh_Goth_ and Lb_16_ (Fig 4) as examples (see results for the other interactions in S2 Fig). In *D*. *suzukii* L1, 90% of the wasp larvae were free 48h post-parasitism, a small percentage being surrounded by a thin coat of lightly colored cells. 72h post-parasitism, a lightly colored cellular coat surrounded almost all larvae, with only 2% of Lh_Goth_ and 5% of Lb_16_ larvae being completely encapsulated. Interestingly, a small percent of Lh_Goth_ infested hosts still hosted free wasp larvae, which roughly corresponds to the success rate when parasitizing *D*. *suzukii* L1. A more rapid immune response occurred in parasitized L2 larvae as most Lh_Goth_ eggs/larvae were surrounded by a cellular coat 48h post-parasitism, but only 7% of larvae contained encapsulated eggs. The percentage of free wasp larvae was also congruent with Lh_Goth_ success rate on *D*. *suzukii* L2. Results were more complex with Lb_16_: 48h post-parasitism, about 70% of L2 hosts contained wasp larvae enclosed in a thin coat of lightly colored cells, 22% showing black melanized spots, while other L2 contained almost equally unhatched parasitoid eggs (typically considered as dead) and free larvae that will be encapsulated later.

## Discussion

Previous investigations on *D*. *suzukii—*parasitoids interactions were obtained mainly by quantifying the final outcome (numbers of emerged non-parasitized adult flies, adult flies with capsules or parasitoids [23–25,27–29]), without monitoring in details the physiological interactions. Here, we sought to better understand the success or failure of parasitism of sympatric and allopatric endoparasitoid wasps on the larval stages of *D*. *suzukii* and *D*. *melanogaster* through qualitative and quantitative analyses of the time course of the parasitoid development and the immune response of the fly larvae. Although the dissection of large numbers of parasitized *D*. *suzukii* larvae is time-consuming due to the low fertility of this species under laboratory conditions [39,40], the data obtained have brought new information compared to the emergence counting approach.

As expected, all parasitoid strains tested oviposited in L1 and L2 larvae of both host species and developed successfully on *D*. *melanogaster* Nasrallah, without being encapsulated or inducing mortality. A more contrasting situation was observed on *D*. *suzukii*. Although the parasitism of *A*. *japonica* induced a 35%-50% host mortality, its development was otherwise successful, consistent with previous laboratory data [28, 29]. However, under our conditions, females had a low propensity to parasitize (about 30%) which surprisingly fell to close to 0% for experienced individuals, suggesting a possible inhibition of the oviposition behavior due to wasp handling. Neither the increase in the duration of parasitism exposure (from 4h to 24h, with a day/night period) nor the use of larger boxes (more space) improved the PP, indicating a possible need for other stimuli, possibly associated with the host-plant interaction, for optimal egg-laying in *D*. *suzukii*. The parasitism of *L*. *heterotoma* (both French and Japanese strains) either induced host mortality or led to the emergence of flies carrying a capsule. However, although infrequent, successful development in *D*. *suzukii* was observed more often than reported by Chabert et al. [27] and at a level of 10–20% similar to that recently described for a European *L*. *heterotoma* field strain. On the other hand, it has been reported that strains Lh_14_ and Lh_sw_ (from California and Sweden) have consistently failed on this species [28]. The success of *L*. *heterotoma* on *D*. *suzukii* thus seems to depend on the parasitoid strain but also possibly on the origin of *D*. *suzukii* and the laboratory conditions. Interestingly, the allopatric *vs* sympatric origin of the parasitoid was not relevant, showing the potential for local *L*. *heterotoma* to develop on a host never encountered. We did not observe an emergence of *L*. *victoriae* on *D*. *suzukii* despite a high infestation rate, especially in the L1 stage, in agreement with the results obtained with two other strains tested [28]. Finally, none of the *L*. *boulardi* strains was able to develop in *D*. *suzukii*, as previously reported [27,28], but they induced a high host mortality. Altogether, under laboratory conditions, *Leptopilina* strains parasitized the invasive Asian fly regardless of their origin and probability of success.

The outcome of the interaction of *D*. *suzukii* with larval parasitoids depends mainly on the physiological adequacy of the parasitoid and its effectiveness to overcome the immune response. All endoparasitoid species encapsulated by *D*. *suzukii* in this study belong to the *Leptopilina* genus. To be successful in their host, these species rely on active immunosuppressive strategies based on the rapid effect of virulence factors present in the venom they inject at oviposition [11,14,17]. However, despite their close relatedness, they differ widely in their virulence properties, venom components and host physiological targets [17,38,41]. It is therefore unlikely that “late” encapsulation in *D*. *suzukii* is mainly related to the effects associated with parasitoid venom. In addition, the medium to high mortality rates of parasitized *D*. *suzukii* larvae suggest that none of the parasitoids tested are truly adapted to this fly. *A*. *japonica* and *L*. *heterotoma*, both considered “generalists” [9,20,42], can produce offspring in *D*. *suzukii* but *L*. *heterotoma* is mainly unable to overcome the encapsulation response, with little variation between strains. Of the *Leptopilina* species, *L*. *boulardi* only develops in a few species of the *D*. *melanogaster* subgroup [43]. Parasitism consistently failed on *D*. *suzukii*, and the late L3 and pupal stages of the parasitized host experienced a high mortality rate. The dead host larvae do not contain capsules, but the parasitoid larvae that have died inside showed important signs of melanization, suggesting a strong incompatibility and an uncontrolled systemic immune response after parasitoid death. Interestingly, the Lbm and Lby strains, which differ in their virulence against *D*. *melanogaster* and *D*. *yakuba* and in their host range [44–45] also behave differently in *D*. *suzukii*. Lbm induces higher host mortality (> 90%) than Lby (70%-75% mortality and 25%-30% of emergence of flies with a capsule), in agreement with its narrower host range.

An intriguing question arose from the observation of the time-course of the interaction: why is the encapsulation of parasitoids systematically "delayed" in *D*. *suzukii*? Indeed, when parasitoid egg encapsulation occurs in *D*. *melanogaster* and in closely-related species, it is completed within 48h of parasitism [11,46–48] (see also Results). The parasitoid egg is killed quickly, usually before the capsule is completely melanized [14]. In contrast, in *D*. *suzukii*, hatched wasp eggs and wasp larvae continue to develop for 72 to 96 hours before dying, and encapsulation/melanisation occurs later. The first premise of encapsulation—the presence of immune cells on the parasitoid—is only detected 72h after parasitism.

In *D*. *melanogaster*, the recognition of a large foreign body such as the wasp egg is followed by a rapid increase in the number of circulating hemocytes, mainly phagocytic plasmatocytes and lamellocytes. Plasmatocytes first attach to the egg chorion, followed by the deposition of several layers of lamellocytes, the main cells involved in the capsule formation [11,12,49]. Lamellocytes differentiate after parasitism and their presence in the hemolymph has two possible origins: sessile hemocyte islets present under the larval cuticle or the lymph gland which is the larval hematopoietic organ [50–53]. In the hemolymph of *D*. *suzukii*, plasmatocyte- and lamellocyte-like cells, as well as larger plasmatocyte type cells called podocytes have been described, and the presence of crystal cells mainly involved in wound healing has been demonstrated [28,29]. The origin of these cells has not yet been studied in *D*. *suzukii*. Although variable, the much higher basal number of these hemocytes in this species than in *D*. *melanogaster* and other *Drosophila* species has been proposed as an explanation for the high resistance of this species to larval endoparasitoid wasps [28,29]. According, only lamellocytes appeared to increase in number in response to an immune challenge in *D*. *suzukii* [28]. Yet, Poyet et al. [29] reported that parasitism of *A*. *japonica* does not cause such an increase in *D*. *suzukii*, unlike that of *L*. *heterotoma*, which could be related to the success of the first species.

Here, we did not measure the lamellocytes number after parasitization. However, interestingly, we did not observe any important accumulation of hemocytes on the surface of parasitoid eggs during the first 48 hours. This suggests that although many plasmatocytes circulate, they may have a poor ability to recognize the egg and adhere to its surface, or that "activated" plasmatocytes with these properties are not yet present in circulation. It is also possible that the eggs of the different species of parasitoids studied, recognized as non-self in *D*. *melanogaster*, are not recognized in *D*. *suzukii* contrary to the larva once hatched. The masking of the egg against host immune cells is used by some species of parasitoids to avoid encapsulation [54] but this strategy has not been reported for the species used here. After hatching, the parasitoid larva would move sufficiently to prevent or further slow adhesion of host hemocytes. Accordingly, we observed a slow and late increase in the number of cells attached to the developing parasitoid, and it was mostly dying or dead larvae that were surrounded by cells.

The lag-time is even more noticeable on the melanization process. In *D*. *melanogaster*, the main source of the phenoloxidase involved in the melanization of the capsule (PPO3) are the lamellocytes [55,56]. In *D*. *suzukii*, the delayed adhesion of plasmatocytes could alter that of the lamellocytes and the subsequent release and activation of the PO. One recognized PO gene (*proPO-A*; DS10_00003111) and several potential others are present in the *D*. *suzukii* genome [57] (http://spottedwingflybase.org). However, there has been no study to assess whether they are equivalent to the three described *D*. *melanogaster* POs and what role(s) they may play in the melanization cascade. The late melanization response may also explain the high level of parasitoid-induced mortality we have recorded, particularly with *L*. *boulardi* and *L*. *japonica* strains, or the occasional parasitic success of *L*. *heterotoma*. It has indeed been reported that when the melanic encapsulation response is not fast or strong enough, the developing wasp can escape the capsule or kill the host [58,59]. At last, the presence of large capsules in adult *D*. *suzukii* flies due to delayed encapsulation could impact their fitness, as reported for *D*. *melanogaster* [60–62], a possible cost that is thus worth to explore.

Our results also question whether local parasitoids could somehow participate in controlling *D*. *suzukii* populations in the newly invaded areas. *L*. *heterotoma* and *L*. *boulardi*, among the most common parasitoid species of *Drosophila* larvae in Europe [63], show a high acceptance level of *D*. *suzukii*. Although *L*. *boulardi* never emerges from *D*. *suzukii*, it could have a significant impact on its populations since it kills 60 to 90% of the larval hosts. However, this effect will be difficult to assess in the field. *L*. *heterotoma* kills about 40% of *D*. *suzukii* larvae and is successful at a low rate in laboratory, which probably accounts for the undetected emergence of this parasitoid from field exposed larvae or sampled pupae in fruits [19,25,64,65]. In semi-natural conditions, we observed that *L*. *heterotoma* could parasitize *D*. *suzukii* within fruits since a low number of flies emerged with a capsule (unpublished observation) and some *D*. *suzukii* flies trapped in the field also harbored a capsule. This suggests that *L*. *heterotoma* may parasitize this fly *in natura* at a low level. Altogether, data suggest that local endoparasitoids may be involved in controlling this new pest by inducing non-reproductive host mortality and reducing fitness if they are able to adapt to its specific niche.

## Supporting information

S1 Fig Outcomes of the interaction between *D*. *suzukii* and parasitoid species/strains. Proportions of death of both partners, emerged wasps, emerged flies with a capsule or without capsule (supposedly non-parasitized) following parasitism of L1 or L2 host larvae by *Asobara japonica* (Aj), *Leptopilina heterotoma* strains from Japan (LhJapan) and South France (Gotheron, LhGoth), the *Leptopilina boulardi* strains ISm (Lbm), ISy (Lby), and Lb16 (field strain)), and a *Leptopilina victoriae* Japanese strain (Lv). CNT: *D*. *suzukii* non-parasitized control flies.
(PDF)

S2 Fig Detailed analysis of the *D*. *suzukii* interaction with the parasitoid species/strains. Pools of 20 *D*. *suzukii* L1 or L2 larvae parasitized either by *Asobara japonica* (Aj), a *Leptopilina heterotoma* Japanese strain (LhJapan), the *Leptopilina boulardi* strains ISm (Lbm) and ISy (Lby), or a *Leptopilina victoriae* Japanese strain (Lv) were dissected 48h (L1 and L2) or 72h (L1) post-parasitism. Pie charts provide the percentage of alive or dead fly larvae containing free parasitoid larvae, free floating eggs, wasp eggs or larvae only surrounded by a thin coat of lightly-colored cells, wasp eggs or larvae partially melanized (coat of lightly-colored cells with a few black spots), and completely encapsulated parasitoid eggs or larvae (fully melanized).
(PDF)

S1 File Summary and results of the selected statistical models. (PDF)

## References

[1] Hauser M. A historic account of the invasion of *Drosophila suzukii* (Matsumura) (Diptera: Drosophilidae) in the continental United States, with remarks on their identification. Pest Manag Sci. 2011;67: 1352–1357. 10.1002/ps.2265 21898759

[2] Calabria G, Máca J, Bächli G, Serra L, Pascual M. First records of the potential pest species *Drosophila suzukii* (Diptera: Drosophilidae) in Europe. J Appl Entomol. 2012;136: 139–147.

[3] Asplen MK, Anfora G, Biondi A, Choi D-S, Chu D, Daane KM, et al Invasion biology of spotted wing Drosophila (*Drosophila suzukii*): a global perspective and future priorities. J Pest Sci. 2015;27: 1–26.

[4] Mitsui H, Takahashi KH, Kimura MT. Spatial distributions and clutch sizes of *Drosophila* species ovipositing on cherry fruits of different stages. Popul Ecol. 2006;48: 233–237.

[5] Atallah J, Teixeira L, Salazar R, Zaragoza G, Kopp A. The making of a pest: the evolution of a fruit-penetrating ovipositor in *Drosophila suzukii* and related species. Proc Biol Sci. 2014;281(1781): 20132840 10.1098/rspb.2013.2840 24573846 PMC3953835

[6] Walsh DB, Bolda MP, Goodhue RE, Dreves AJ, Lee J, Bruck DJ. *Drosophila suzukii* (Diptera: Drosophilidae): invasive pest of ripening soft fruit expanding its geographic range and damage potential. J Integr Pest Manag. 2011;2(1): 1–7.

[7] Poyet M, Le Roux V, Gibert P, Meirland A, Prevost G, Eslin P, et al The wide potential trophic niche of the Asiatic fruit fly *Drosophila suzukii*: The key of its invasion success in yemperate Europe? PLoS One. 2015;10(11): e0142785 10.1371/journal.pone.0142785 26581101 PMC4651357

[8] Kenis M, Tonina L, Eschen R, van der Sluis B, Sancassani M, Mori N, et al Non-crop plants used as hosts by *Drosophila suzukii* in Europe. J Pest Sci. 2016;89: 735–748. 10.1007/s10340-016-0755-6 PMC5318492 28275324

[9] Carton Y, Boulétreau M, van Alphen JJM, van Lenteren JC. The *Drosophila* parasitic wasps In: Ashburner M, Carson L, Thompson JN, editors. The Genetics and Biology of *Drosophila*. London: Academic Press; 1986 pp. 347–394.

[10] Godfray HCJ. Parasitoids: Behavioral and evolutionary ecology. Princeton: Princeton University Press; 1994.

[11] Carton Y, Poirié M, Nappi AJ. Insect immune resistance to parasitoids. Insect Sci. 2008;15: 67–87.

[12] Fauvarque M-O, Williams MJ. *Drosophila* cellular immunity: a story of migration and adhesion. J Cell Sci. 2011;124: 1373–1382. 10.1242/jcs.064592 21502134

[13] Rivers DB, Ruggiero L, Hayes M. The ectoparasitic wasp *Nasonia vitripennis* (Walker) (Hymenoptera: Pteromalidae) differentially affects cells mediating the immune response of its flesh fly host, *Sarcophaga bullata* Parker (Diptera: Sarcophagidae). J Insect Physiol 2002;48:1053–64. 12770028 10.1016/s0022-1910(02)00193-2

[14] Poirié M, Carton Y, Dubuffet A. Virulence strategies in parasitoid Hymenoptera as an example of adaptive diversity. C R Biol. 2009;332: 311–320. 10.1016/j.crvi.2008.09.004 19281961

[15] Goecks J, Mortimer NT, Mobley JA, Bowersock GJ, Taylor J, Schlenke TA. Integrative Approach Reveals Composition of Endoparasitoid Wasp Venoms. PLoS One. 2013;8: e64125 10.1371/journal.pone.0064125 23717546 PMC3662768

[16] Keebaugh ES, Schlenke TA. Insights from natural host-parasite interactions: The *Drosophila* model. Dev Comp Immunol. 2013;42: 111–123. 10.1016/j.dci.2013.06.001 23764256 PMC3808516

[17] Poirié M, Colinet D, Gatti J-L. Insights into function and evolution of parasitoid wasp venoms. Curr Opin Insect Sci. 2014;6: 52–60. 10.1016/j.cois.2014.10.004 32846678

[18] Kanzawa T. Studies on *Drosophila suzukii* Mats. Yamanashi Agricultural Experiment Station, Kofu, Japan; 1939. [English abstract in: Rev Appl Entomol. 29: 622].

[19] Mitsui H, Van Achterberg K, Nordlander G, Kimura MT. Geographical distributions and host associations of larval parasitoids of frugivorous Drosophilidae in Japan. J Nat Hist. 2007;41: 1731–1738.

[20] Ideo S, Watada M, Mitsui H, Kimura MT. Host range of *Asobara japonica* (Hymenoptera: Braconidae), a larval parasitoid of Drosophilid flies. Entomol Sci. 2008;11: 1–6.

[21] Mitsui H, Kimura MT. Distribution, abundance and host association of two parasitoid species attacking frugivorous drosophilid larvae in central Japan. Eur J Entomol. 2010;107: 535–540.

[22] Novković B, Mitsui H, Suwito A, Kimura MT. Taxonomy and phylogeny of *Leptopilina* species (Hymenoptera: Cynipoidea: Figitidae) attacking frugivorous drosophilid flies in Japan, with description of three new species. Entomol Sci. 2011;14: 333–346.

[23] Kasuya N, Mitsui H, Ideo S, Watada M, Kimura MT. Ecological, morphological and molecular studies on *Ganaspis* individuals (Hymenoptera: Figitidae) attacking *Drosophila suzukii* (Diptera: Drosophilidae). Appl Entomol Zool. 2013;48: 87–92.

[24] Nomano FY, Kasuya N, Matsuura A, Suwito A, Mitsui H, Buffington ML, et al Genetic differentiation of *Ganaspis brasiliensis* (Hymenoptera: Figitidae) from East and Southeast Asia. Appl Entomol Zool. 2017;63: 742–749.

[25] Girod P, Rossignaud L, Haye T, Turlings TCJ, Kenis M. Development of Asian parasitoids in larvae of *Drosophila suzukii* feeding on blueberry and artificial diet. J Appl Entomol. 2018;88: 1–12.

[26] Wang X-G, Nance AH, Jones JML, Hoelmer KA, Daane KM. Aspects of the biology and reproductive strategy of two Asian larval parasitoids evaluated for classical biological control of *Drosophila suzukii*. Biol Control 2018;121: 58–65.

[27] Chabert S, Allemand R, Poyet M, Eslin P, Gibert P. Ability of European parasitoids (Hymenoptera) to control a new invasive Asiatic pest, *Drosophila suzukii*. Biol Control. 2012;63: 40–47.

[28] Kacsoh BZ, Schlenke TA. High hemocyte load is associated with increased resistance against parasitoids in *Drosophila suzukii*, a relative of *D*. *melanogaster*. PLoS One. 2012;7(4): e34721 10.1371/journal.pone.0034721 22529929 PMC3328493

[29] Poyet M, Havard S, Prevost G, Chabrerie O, Doury G, Gibert P, et al Resistance of *Drosophila suzukii* to the larval parasitoids *Leptopilina heterotoma* and *Asobara japonica* is related to haemocyte load. Physiol Entomol. 2013;38: 45–53.

[30] Rossi-Stacconi MV, Grassi A, Dalton DT, Miller B, Ouantar M, Loni A, et al First field records of *Pachycrepoideus vindemiae* as a parasitoid of *Drosophila suzukii* in European and Oregon small fruit production areas. Entomologia. 2013;1: 11–16.

[31] Rossi-Stacconi MV, Amiresmaeili N, Biondi A, Carli C, Caruso S, Dindo ML, et al Host location and dispersal ability of the cosmopolitan parasitoid *Trichopria drosophilae* released to control the invasive spotted wing *Drosophila*. Biol Control 2018;117: 188–196.

[32] Wang X-G, Kaçar G, Biondi A, Daane KM. Foraging efficiency and outcomes of interactions of two pupal parasitoids attacking the invasive spotted wing drosophila. Biol Control. 2016;96: 64–71.

[33] Eslin P, Prevost G. Hemocyte load and immune resistance to *Asobara tabida* are correlated in species of the *Drosophila melanogaster* subgroup. J Insect Physiol. 1998;44: 807–816. 12769876 10.1016/s0022-1910(98)00013-4

[34] Carton Y, Frey F, Nappi AJ. Genetic determinism of the cellular immune reaction in *Drosophila melanogaster*. Heredity. 1992;69: 393–399. 1428954 10.1038/hdy.1992.141

[35] Dupas S, Brehélin M, Frey F, Carton Y. Immune suppressive virus-like particles in a *Drosophila* parasitoid: significance of their intraspecific morphological variations. Parasitology. 1996;113(13): 207–212. 8811846 10.1017/s0031182000081981

[36] Lin QC, Zhai Y-F, Zhang AF, Men X-Y, Zhang X-Y, Zalom FG, et al Comparative Developmental Times and Laboratory Life Tables for *Drosophila suzukii* and *Drosophila melanogaster* (Diptera: Drosophilidae). Fla Entomol 2014;97(4): 1434–1442.

[37] Ris N, Allemand R, Fouillet P., Fleury F. The joint effect of temperature and host species induce complex genotype-by-environment interactions in the larval parasitoid of *Drosophila*, *Leptopilina* heterotoma (Hymenoptera: Figitidae). Oikos. 2004;106: 451–456.

[38] Dubuffet A, Colinet D, Anselme C, Dupas S, Carton Y, Poirié M. Variation of *Leptopilina boulardi* success in *Drosophila* hosts: what is inside the black box? Adv Parasitol. 2009;70: 147–188. 10.1016/S0065-308X(09)70006-5 19773070

[39] Emiljanowicz LM, Ryan GD, Langille A, Newman J. Development, Reproductive Output and Population Growth of the Fruit Fly Pest *Drosophila suzukii* (Diptera: Drosophilidae) on Artificial Diet. J Econ Entomol. 2014; 107(4): 1392–1398. 25195427 10.1603/ec13504

[40] Iacovone A, Girod P, Ris N, Weydert C, Gibert P, Poirié M, et al Worldwide invasion by *Drosophila suzukii*: Does being the “cousin” of a model organism really help setting up biological control? Hopes, disenchantments and new perspectives. Rev Ecol Terre Vie 2015;70: 207–214.

[41] Moreau S, Asgari S. Venom Proteins from Parasitoid Wasps and Their Biological Functions. Toxins (Basel) 2015;7: 2385–2412. 26131769 10.3390/toxins7072385 PMC4516919

[42] Jenni W. Beitrag zur morphologie und biologie der cynipide *Pseudeucoila bochei* weld, eines larvenparasiten von *Drosophila melanogaster* meig. Acta zool. 1951;32(3): 177–254.

[43] Carton Y, Kitano H. Evolutionary relationships to parasitism by seven species of the *Drosophila melanogaster* subgroup. Biol J Linn Soc Lond. 1981;16: 227–241.

[44] Dupas S, Carton Y, Poirié M. Genetic dimension of the coevolution of virulence-resistance in *Drosophila*—parasitoid wasp relationships. Heredity 2003;90: 84–89. 10.1038/sj.hdy.6800182 12522430

[45] Dupas S, Poirié M, Frey F, Carton Y. Is parasitoid virulence against multiple hosts adaptive or constrained by phylogeny? A study of *Leptopilina* spp. (Hymenoptera: Figitidae)/*Drosophila* (Diptera: Drosophilidae) interactions. Ann Soc Entomol Fr. 2013;49: 222–231.

[46] Nappi AJ, Streams FA. Abortive development of the cynipid parasite *Pseudeucoila bochei* (Hymenoptera) in species of the *Drosophila melanica* group. Ann Entomol Soc Am. 1970;63(1): 321–327.

[47] Carton Y, Nappi AJ. *Drosophila* cellular immunity against parasitoids. Parasitol Today. 1997;13: 218–227. 15275074 10.1016/s0169-4758(97)01058-2

[48] Dubuffet A, Doury G, Labrousse C, Drezen J-M, Carton Y, Poirié M. Variation of success of *Leptopilina boulardi* in *Drosophila yakuba*: the mechanisms explored. Dev Comp Immunol. 2008;32(6): 597–602. 10.1016/j.dci.2007.10.009 18061668

[49] Rizki TM, Rizki RM. Lamellocyte differentiation in *Drosophila* larvae parasitized by *Leptopilina*. Dev Comp Immunol. 1992;16: 103–110. 1499832 10.1016/0145-305x(92)90011-z

[50] Stofanko M, Kwon SY, Badenhorst P. Lineage Tracing of Lamellocytes Demonstrates *Drosophila* Macrophage Plasticity. PLoS One. 2010;5: e14051 10.1371/journal.pone.0014051 21124962 PMC2988793

[51] Krzemien J, Oyallon J, Crozatier M, Vincent A. Hematopoietic progenitors and hemocyte lineages in the *Drosophila* lymph gland. Dev Biol. 2010;346(2): 310–319. 10.1016/j.ydbio.2010.08.003 20707995

[52] Honti V, Csordás G, Kurucz E, Márkus R, Andó I. The cell-mediated immunity of *Drosophila melanogaster*: hemocyte lineages, immune compartments, microanatomy and regulation. Dev Comp Immunol. 2014;42: 47–56. 10.1016/j.dci.2013.06.005 23800719

[53] Anderl I, Vesala L, Ihalainen TO, Vanha-aho L-M, Andó I, Rämet M, et al Transdifferentiation and Proliferation in Two Distinct Hemocyte Lineages in *Drosophila melanogaster* Larvae after Wasp Infection. PLoS Pathog. 2016;12: e1005746–34. 10.1371/journal.ppat.1005746 27414410 PMC4945071

[54] Eslin P, Prevost G. Racing against host’s immunity defenses: a likely strategy for passive evasion of encapsulation in *Asobara tabida* parasitoids. J Insect Physiol. 2000;46: 1161–1167. 10818243 10.1016/s0022-1910(99)00227-9

[55] Binggeli O, Neyen C, Poidevin M, Lemaitre B. Prophenoloxidase Activation Is Required for Survival to Microbial Infections in *Drosophila*. PLoS Pathog. 2014;10: e1004067 10.1371/journal.ppat.1004067 24788090 PMC4006879

[56] Dudzic JP, Kondo S, Ueda R, Bergman CM, Lemaitre B. *Drosophila* innate immunity: regional and functional specialization of prophenoloxidases. BMC Biol. 2015;13: 1–16. 26437768 10.1186/s12915-015-0193-6 PMC4595066

[57] Chiu JC, Jiang X, Zhao L, Hamm CA, Cridland JM, Saelao P, et al Genome of *Drosophila suzukii*, the Spotted Wing Drosophila. G3 (Bethesda). 2013;3(12): 2257–2271. 24142924 10.1534/g3.113.008185 PMC3852387

[58] Strand MR, Pech LL. Immunological basis for compatibility in parasitoid-host relationships. Annu Rev Entomol. 1995;40: 31–56. 10.1146/annurev.en.40.010195.000335 7810989

[59] Salazar-Jaramillo L, Paspati A, De Zande Van L, Vermeulen CJ, Schwander T, Wertheim B. Evolution of a Cellular Immune Response in *Drosophila*: A Phenotypic and Genomic Comparative Analysis. Genome Biol Evol. 2014;6: 273–289. 10.1093/gbe/evu012 24443439 PMC3942026

[60] Carton Y, David JR. Reduction of fitness in *Drosophila* adults surviving parasitism by a cynipid wasp. Experientia. 1983;39: 231–233.

[61] Fellowes MDE, Kraaijeveld AR, Godfray HCJ. The relative fitness of *Drosophila melanogaster* (Diptera, Drosophilidae) that have successfully defended themselves against the parasitoid *Asobara tabida* (Hymenoptera, Braconidae). J Evol Biol. 1999;12: 123–128.

[62] Kraaijeveld AR, Ferrari J, Godfray HCJ. Costs of resistance in insect-parasite and insect-parasitoid interactions. Parasitology. 2002;125: S71–S82. 12622330 10.1017/s0031182002001750

[63] Allemand R, Fleury F, Lemaitre C, Boulétreau M. Population dynamics and competitive interactions in two species of *Leptopilina* (Hymenoptera: Figitidae) which parasitize *Drosophila* in the Rhône valley (S-E France). Ann Soc Entomol Fr. 1999;35: 97–103.

[64] Cini A, Anfora G, Escudero-Colomar LA, Grassi A. Tracking the invasion of the alien fruit pest *Drosophila suzukii* in Europe. J Pest Sci. 2014;87(4): 559–566.

[65] Kremmer L, Thaon M, Borowiec N, David J, Poirié M, Gatti J-L, et al Field Monitoring of *Drosophila suzukii* and Associated Communities in South Eastern France as a Pre-Requisite for Classical Biological Control. Insects 2017;8(4): e124 10.3390/insects8040124 29144440 PMC5746807

