# Supplementary figures and images for: Time-course analysis of Drosophila suzukii interaction with endoparasitoid wasps evidences a delayed encapsulation response compared to D. melanogaster
Source: PLoS One. 2018 Aug 2;13(8):e0201573. doi: 10.1371/journal.pone.0201573 (PMC6072091; doi:10.1371/journal.pone.0201573)

### Infection outcome: *D. suzukii* L1

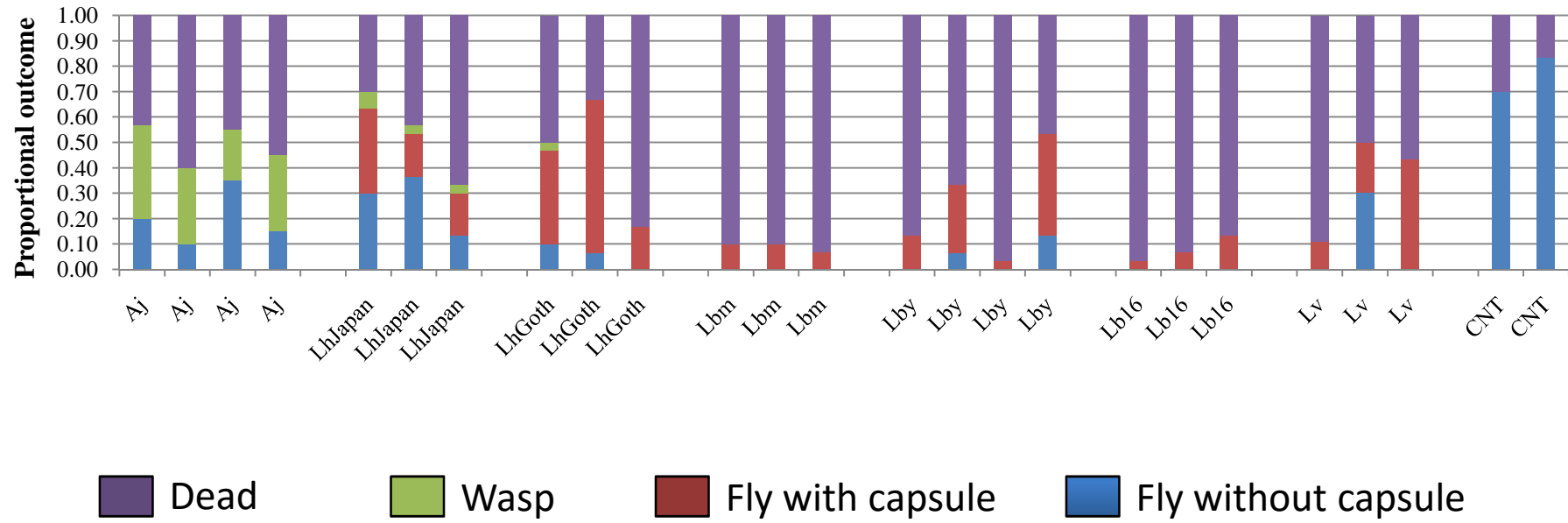

### Infection outcome: *D. suzukii* L2

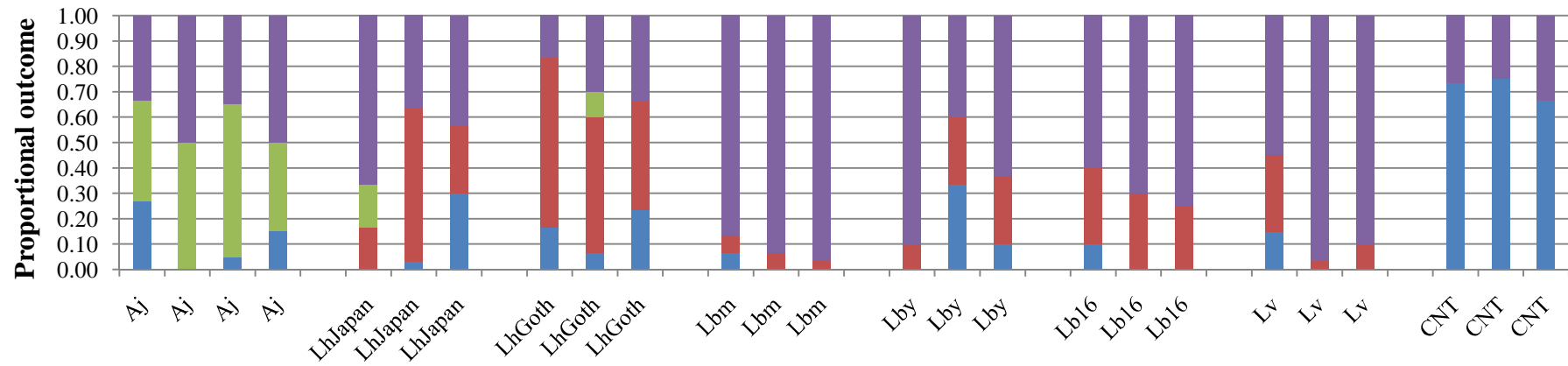

Supplement: S1 Fig — Proportions of death of both partners, emerged wasps, emerged flies with a capsule or without capsule (supposedly non-parasitized) following parasitism of L1 or L2 host larvae by Asobara japonica (Aj), Leptopilina heterotoma strains from Japan (LhJapan) and South France (Gotheron, LhGoth), the Leptopilina boulardi strains ISm (Lbm), ISy (Lby), and Lb16 (field strain)), and a Leptopilina victoriae Japanese strain (Lv). CNT: D. suzukii non-parasitized control flies. (PDF) [file pone.0201573.s001.pdf]
