# Supplementary material for: Time-course analysis of Drosophila suzukii interaction with endoparasitoid wasps evidences a delayed encapsulation response compared to D. melanogaster
Source: PLoS One. 2018 Aug 2;13(8):e0201573. doi: 10.1371/journal.pone.0201573 (PMC6072091; doi:10.1371/journal.pone.0201573)
Supplement: S2 Fig — Pools of 20 D. suzukii L1 or L2 larvae parasitized either by Asobara japonica (Aj), a Leptopilina heterotoma Japanese strain (LhJapan), the Leptopilina boulardi strains ISm (Lbm) and ISy (Lby), or a Leptopilina victoriae Japanese strain (Lv) were dissected 48h (L1 and L2) or 72h (L1) post-parasitism. Pie charts provide the percentage of alive or dead fly larvae containing free parasitoid larvae, free floating eggs, wasp eggs or larvae only surrounded by a thin coat of lightly-colored cells, wasp eggs or larvae partially melanized (coat of lightly-colored cells with a few black spots), and completely encapsulated parasitoid eggs or larvae (fully melanized). (PDF) [file pone.0201573.s002.pdf]

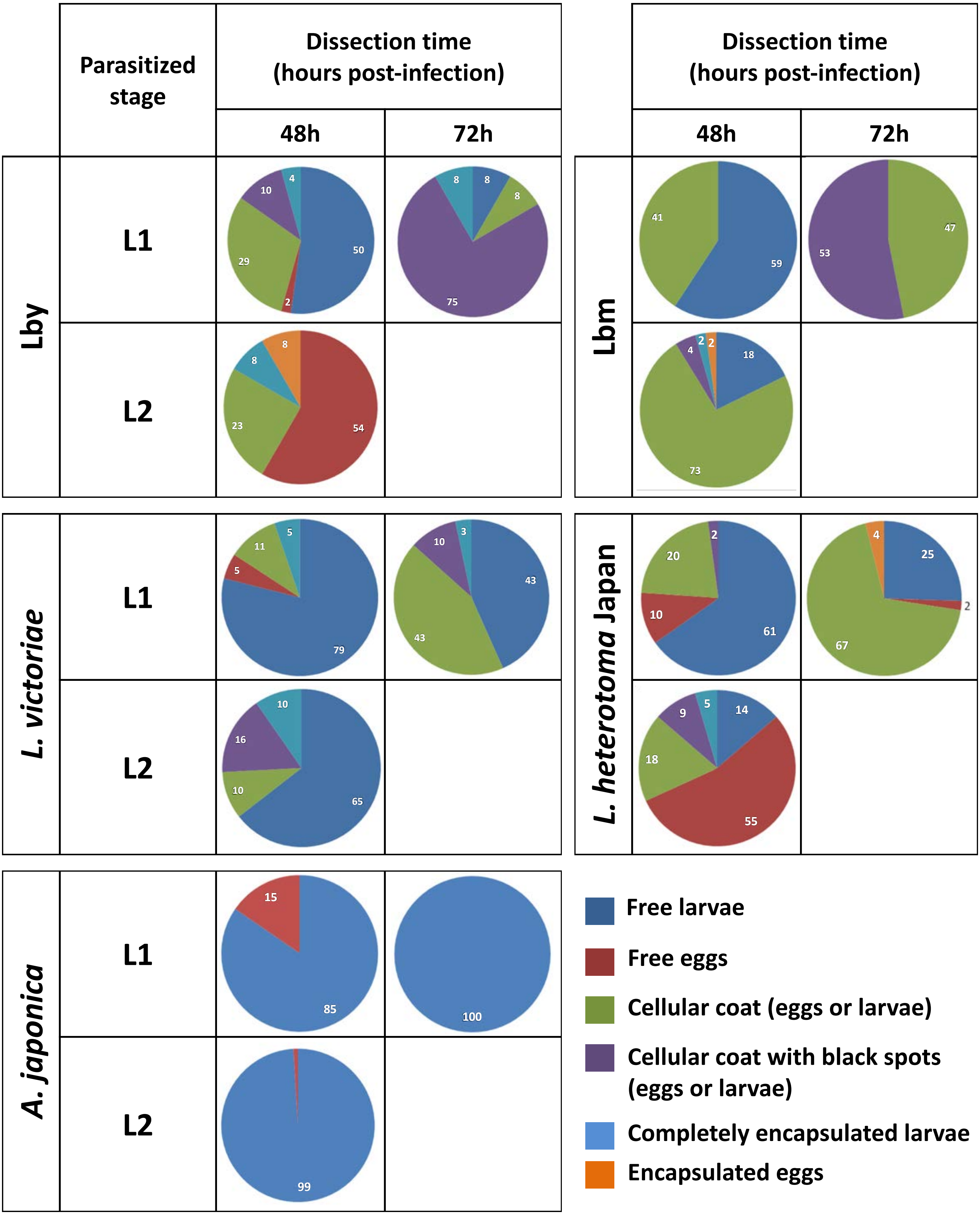

Supplementary Figure : Outcome of the parasitoid in parasitized *D. suzukii* L1 (48/72h post-infection) and L2 larvae (48h post-infection). Numbers in percent.
