# Supplementary material for: Time-course analysis of Drosophila suzukii interaction with endoparasitoid wasps evidences a delayed encapsulation response compared to D. melanogaster
Source: PLoS One. 2018 Aug 2;13(8):e0201573. doi: 10.1371/journal.pone.0201573 (PMC6072091; doi:10.1371/journal.pone.0201573)
Supplement: S1 File — (PDF) [file pone.0201573.s003.pdf]

Supplement 1:

Summary of the selected statistical models

Traits	Factors	X^2	df	p-value
Propensity to parasitize				
**Null Model: Deviance: 143.0; df =55**				
**Selected Model: Residual Deviance: 59.4; df=47**				
**AIC: 121,3**				
	**Parasitoid strain**	**69.9**	**6**	$p<10^{-3}$
	**Host species**	**6.9**	**1**	**0.008**
	**Experience**	**2.6**	**1**	**0.108**
Infestation rate– All parasitoid strains				
**Null Model: Deviance: 839.7; df =126**				
**Selected Model: Residual Deviance: 502.1; df=99**				
**AIC: 918.6**				
	**Parasitoid strain (P)**	**132.8**	**6**	$p<10^{-3}$
	**Host Species (H)**	**70.5**	**1**	$p<10^{-3}$
	**Host Stage (S)**	**0.6**	**1**	**0.422**
	**P x H**	**42.3**	**6**	$p<10^{-3}$
	**P x S**	**50.5**	**6**	$p<10^{-3}$
	**H x S**	**1.4**	**1**	**0.237**
	**P x H x S**	**48.1**	**6**	$p<10^{-3}$
Infestation rate – Japanese strains only				
**Null Model: Deviance: 358.8; df =55**				

Selected Model: Residual Deviance: 258.7; df=44

AIC: 444

**Parasitoid strain (P)**	**28.8**	**2**	**$p<10^{-3}$**
**Host Species (H)**	**23.6**	**1**	**$p<10^{-3}$**
**Host Stage (S)**	**8.5**	**1**	**0.003**
**P x H**	**6.7**	**2**	**0.04**
**P x S**	**19.6**	**2**	**$p<10^{-3}$**
**H x S**	**0.3**	**1**	**0.61**
**P x H x S**	**9.4**	**2**	**0.009**

***Ability to develop in* D. suzukii**

Null Model: Deviance: 44.3; df =13

Selected Model: Residual Deviance: 3.8; df=7

AIC: 22.7

**Parasitoid strain (P)**	**40.5**	**6**	**$p<10^{-3}$**

Sensitivity to encapsulation by D. suzukii

Null Model: Deviance: 44.6; df =13

Selected Model: Residual Deviance: 0.0; df=7

AIC: 17.2

**Parasitoid strain (P)**	**44.6**	**6**	**$p<10^{-3}$**

***Proportion of encapsulated larvae* (*Asobara japonica* discarded)**

Null Model: Deviance: 237.6; df =36

Selected Model: Residual Deviance: 117.1; df=30

AIC: 244.7

**Parasitoid (P)**	**114.5**	**5**	**$p<10^{-3}$**
**Host Stage (H)**	**5.7**	**1**	**0.02**

Table 1: Pairwise comparisons between the propensity to parasitize and the encapsulation rate for the different parasitoid strains.

	Lv	Lh Goth	Lh Japan	Lbm	Lby	L16
Aj	|z| = 2.50 p = 0.14	|z| = 4.61 $p < 10^{-3}$	|z| = 4.46 $p < 10^{-3}$	|z| = 4.44 $p < 10^{-3}$	|z| = 3.74 p = 0.003	|z| = 3.69 p = 0.004
Lv		|z| = 2.93 p = 0.05	|z| = 2.17 p = 0.29	|z| = 3.02 p = 0.04	|z| = 1.53 p = 0.70	|z| = 0.33 p = 1.00
Lh Goth	|z| = 1.52 p = 0.64		|z| = 1.30 p = 0.84	|z| = 0.42 p = 1.00	|z| = 1.66 p = 0.62	|z| = 0.68 p = 0.99
Lh Japan	|z| = 0.63 p = 0.99	|z| = 0.78 p = 0.97		|z| = 1.61 p = 0.65	|z| = 0.51 p = 1.00	|z| = 1.59 p = 0.66
Lbm	|z| = 3.23 p = 0.015	|z| = 4.54 $p < 10^{-3}$	|z| = 3.65 p = 0.004		|z| = 1.92 p = 0.44	|z| = 0.33 p = 1.00
Lby	|z| = 1.08 p = 0.89	|z| = 2.77 p = 0.06	|z| = 1.69 p = 0.53	|z| = 2.49 p = 0.12		|z| = 1.84 p = 0.49
L16	|z| = 3.40 p =0.008	|z| = 4.66 $p < 10^{-3}$	|z| = 3.81 p = 0.002	|z| = 0.26 p =1.00	|z| = 2.69 p = 0.07	

Above the diagonal: Propensity to parasitize

Below the diagonal: Encapsulation rate (*Asobara japonica* was discarded because of total absence of encapsulation)
